# Global trends of *Pseudomonas aeruginosa* biofilm research in the past two decades: A bibliometric study

**DOI:** 10.1002/mbo3.1021

**Published:** 2020-03-02

**Authors:** Yuanyuan Zhu, Jiao Jiao Li, Jian Reng, Siyang Wang, Ruiqing Zhang, Bin Wang

**Affiliations:** ^1^ Department of Pharmacy Shanxi Medical University Second Affiliated Hospital Taiyuan China; ^2^ Kolling Institute University of Sydney Sydney NSW Australia; ^3^ Department of Orthopaedic Shanxi Medical University Second Affiliated Hospital Taiyuan China

**Keywords:** bibliometric analysis, global trends, *Pseudomonas aeruginosa* biofilm, visualized analysis

## Abstract

*Pseudomonas aeruginosa* biofilm formation is a primary cause of chronic infections. This has been a highly active area of research over the past two decades due to causing high mortality risks in immunocompromised patients. This study evaluates global trends in the dynamic and rapidly evolving field of *P. aeruginosa* biofilm research through bibliometric and visualized analyses. Publications from 1994 to 2018 on *P. aeruginosa* biofilm research were retrieved from Web of Science, Scopus, and PubMed, and their bibliometric data were systematically studied. The VOSviewer software was used to conduct global analyses of bibliographic coupling, coauthorship, cocitation, and co‐occurrence. A total of 9,527 publications were included in this study. The overall number of publications and research interest in the field displayed a strongly rising trend. The USA made the greatest contributions to the field, with the highest h‐index and number of citations compared with other countries, while Denmark had the highest average citation per publication. The *Journal of Bacteriology* had the highest number of publications in the field, while the University of Copenhagen was the institution with the highest contribution influence. Co‐occurrence network maps revealed that the most prominent topics in *P. aeruginosa* biofilm research were mechanistic studies, in vitro/in vivo studies, and biofilm formation studies. *Pseudomonas aeruginosa* biofilms constitute a dynamic research area in microbiology with increasing global research interest. Future studies will likely focus on investigating the mechanisms of biofilm formation to solve infection‐associated clinical problems.

## Graphical abstract

*Pseudomonas aeruginosa* biofilm formation is a primary cause of chronic infections and has been a highly active area of research over the past two decades. This study evaluates global trends in the dynamic and rapidly evolving field of *P. aeruginosa* biofilm research through bibliometric and visualized analyses.

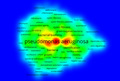

## 1 INTRODUCTION

*Pseudomonas aeruginosa* is a gram‐negative bacterium commonly found in soil and water (Rahme et al., 1995). It is an opportunistic pathogen capable of causing various infections, particularly in immunocompromised or critically ill patients, including bacteremia, sepsis, pneumonia, and skin and wound infections (Kipnis, Sawa, & Wiener‐Kronish, 2006). Infections caused by *P. aeruginosa* can be very serious and life‐threatening, particularly with the emergence of drug‐resistant strains (Zowawi et al., 2015). Antimicrobial resistance is one of the biggest threats to human and animal health today. This has caused widespread concern since *P. aeruginosa* is one of the most prevalent nosocomial pathogens, responsible for 57% of total hospital‐acquired infections (Appanna, Sarabhai, Sharma, & Capalash, 2013).

Biofilm formation is a primary characteristic of *P. aeruginosa* chronic infections (Bjarnsholt, 2013). A biofilm is a community of bacteria attached to a substratum or surface (Chen, Yu, & Sun, 2013), typically consisting of densely packed, multi‐species populations of cells. The main feature of biofilms is the presence of highly hydrated extracellular polymeric substance (EPS), including polysaccharides, proteins, and extracellular DNA (eDNA) (Billings, Birjiniuk, Samad, Doyle, & Ribbeck, 2015). *Pseudomonas aeruginosa* biofilms are generated through a process whereby the bacterial cells are surrounded to form an aggregated structure, which exhibits increased resistance to antibiotics and other anti‐infection agents (Head & Yu, 2003; Mah et al., 2003; Rybtke, Hultqvist, Givskov, & Tolker‐Nielsen, 2015; Whiteley et al., 2001). Therefore, infections caused by biofilm‐forming *P. aeruginosa*, such as in cystic fibrosis of the lung, are almost impossible to eradicate (Guo et al., 2014), and additional challenges are encountered when treating infections caused by multi‐drug‐resistant strains. These complications lead to increased patient morbidity and mortality, higher costs of treatment, and greater rates and time of hospitalization (Costerton, Stewart, & Greenberg, 1999; Lister, Wolter, & Hanson, 2009). Diverse strategies are being pursued to develop novel agents that can kill (new antibiotics) or disarm (antivirulence) the pathogen (Bassetti, Vena, Croxatto, Righi, & Guery, 2018).

In the context of dynamic changes in the field of *P. aeruginosa* biofilm research, it becomes particularly interesting to understand the most important contributions to the field and to predict future research trends. In this study, a bibliometric analysis was conducted on publications in the field of *P. aeruginosa* biofilm research to reflect the global state of the field over the past two decades. This type of analysis can qualitatively and quantitatively evaluate the trends within a particular research community over a defined time frame. It also provides a neat avenue for predicting the main direction of future research in the field, drawing on the relative contributions of different journals, research groups, institutes, and countries (Bassetti et al., 2018). In recent years, bibliometric analysis has been successfully applied in a range of research areas to increase the transparency of published studies (Jia et al., 2015; Wang, Xing, Zhu, Dong, & Zhao, 2019), as well as to assist the development of clinical policies and guidelines (Tacconelli et al., 2018).

Bibliometric analysis has not been previously applied to the field of *P. aeruginosa* biofilm research to investigate the quality and quantity of published studies. The importance of this field is reflected by the recent listing of carbapenem‐resistant *P. aeruginosa* by the World Health Organization (WHO) as one of three bacterial species for which there is a critical need for the development of new antibiotics to treat infections (Tacconelli et al., 2018). Our study, therefore, provides a timely analysis of the overall status of *P. aeruginosa* biofilm research, revealing trends that would be useful for gaining a broad understanding of global developments in the field and future directions.

## 2 MATERIALS AND METHODS

### 2.1 Data source

The Web of Science (WoS) was used for retrieving publication data due to its ability to provide a comprehensive citation search across multiple databases containing cross‐disciplinary research studies. Bibliometric analysis was applied to the publication data. Scopus and PubMed databases were also searched to complement the findings.

### 2.2 Search strategy

WoS was used to retrieve the publication data, with the date range set between 1 January 1994 and 30 December 2018. The search terms used were theme = *Pseudomonas aeruginosa* biofilm OR Pseudomonas pyocyanea biofilm AND publishing year = (1994–2018) AND Language = (English) AND Document types **=** (REVIEW OR ARTICLE). Publication data for specific countries or regions were extracted by refining the search after selecting the appropriate country/region in WoS. For Scopus and PubMed, the search terms were (“*Pseudomonas aeruginosa* biofilm” [Mesh Terms] OR “Pseudomonas pyocyanea biofilm” [All Fields]) AND (“1994/01/01” [PDAT]: “2018/12/31” [PDAT]).

### 2.3 Data collection

The full records of each publication retrieved in the search, including author names, nationalities and affiliations, article title, year of publication, name of publishing journal, keywords, and abstract, were downloaded as a.txt file from the WoS, Scopus, and PubMed databases and subsequently imported into Excel 2016. Two authors (YZ and BW) independently extracted relevant data from these publications. Any disagreement was resolved by discussion or seeking assistance from an external expert to reach consensus. The data were then analyzed independently by the two authors using GraphPad Prism 7.

### 2.4 Bibliometric analysis

The basic characteristics of retrieved publications, including the total number and number by country, total and average citation frequency by country, and H‐index, were described using the intrinsic functions of WoS. The H‐index was used to measure the impact of scientific research arising from different countries within the chosen field of study. The H‐index assigns a value based on an H number of published papers, each of which has been cited by other published papers at least H times. The H‐index for a particular country is, therefore, a reflection of research impact, incorporating both the total number of publications and the average number of citations per publication.

### 2.5 Visualized analysis

VOSviewer (Leiden University, Leiden, The Netherlands) was used for constructing and visualizing the bibliometric networks in this study. Networks were constructed based on bibliographic coupling, coauthorship, and cocitation analysis, using the information on authors, journals, institutions, and countries associated with the publications. Co‐occurrence networks were also constructed to allow visualization of important scientific terms arising from the included publications.

## 3 RESULTS

### 3.1 Trends in global publications

#### 3.1.1 Global publications by country

**Figure 1:**
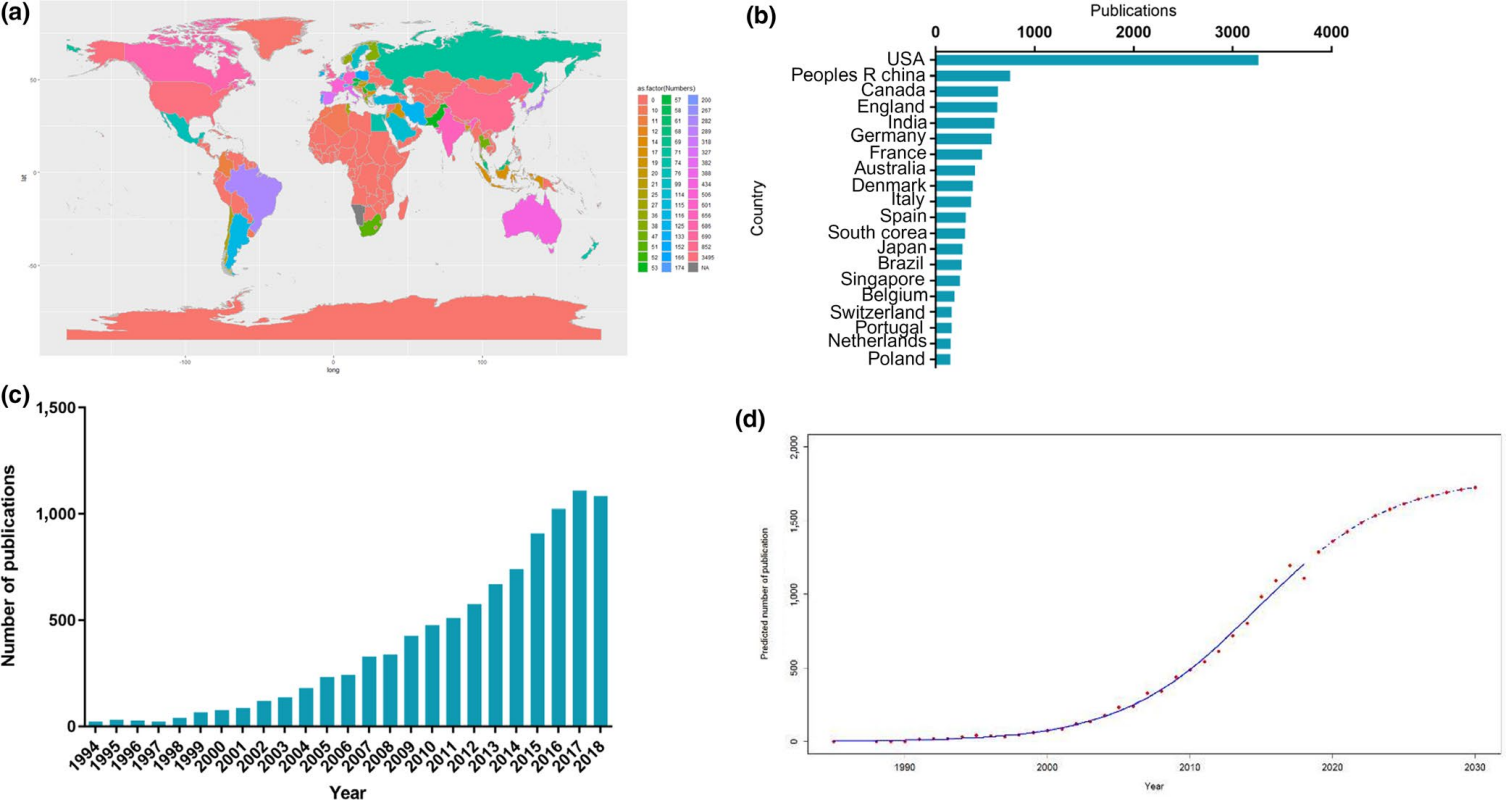
Global trends and countries contributing to *Pseudomonas aeruginosa* biofilm research. (a) World map showing the distribution of publications on *Pseudomonas aeruginosa* biofilm research. (b) The sum of related articles from the top 20 countries. (c) The single‐year publication numbers in the past 24 years. (d) Model fitting curves of growth trends in worldwide publications and prediction of future publication numbers

A total of 9,527 articles from 1994 to 2018 in the field of *P. aeruginosa* biofilm research met the search criteria in WoS. Global contributions by the number of publications to the field are represented on a colored map, grouped by country (Figure 1a). The USA published the highest number of articles in the field (3,261; 34.23%), followed by China (752; 7.89%), England, (628; 6.59%), Canada (624; 6.55%), and India (593; 6.22%) (Figure 1b). Within each of the top 10 contributing countries, a list of the three institutions with the highest number of publications in the field is presented in Table 1. The total number of articles in the field from Scopus and PubMed was 7,280 and 5,206, respectively, all of which were covered by the WoS search. The WoS search results were used for all subsequent bibliographic coupling, coauthorship, cocitation, and co‐occurrence analyses using VOSviewer.

**Table 1:** Institutions with the highest number of publications on *Pseudomonas aeruginosa* biofilms research within each of the top 10 contributing countries

Country	Top contributing institutions	Number of publications
USA	Montana State University	219
	University of Washington	158
	Harvard University	147
China	Chinese Academy of Sciences	114
	Nankai University	41
	Shandong University	21
Canada	University of Calgary	96
	University of Toronto	84
	University of Guelph	57
England	University of Nottingham	78
	University of Oxford	49
	University of London Imperial College of Science and Technology (now Imperial College London)	41
India	Alagappa University	61
	Aligarh Muslim University	39
	Panjab University	39
Germany	Helmholtz Centre for Infection Research	72
	Hannover Medical School	45
	Technical University of Munich (TUM)	32
France	The National Council for Scientific Research (CNRS)	76
	Pasteur Institute	51
	University of Rouen	44
Australia	University of New South Wales	108
	University of Technology Sydney	39
	University of Sydney	37
Denmark	University of Copenhagen	214
	Technical University of Denmark	166
	Rigshospitalet	86
Italy	University of Milan	43
	University of Rome	43
	University of Pisa	23

#### 3.1.2 Global publications by year

The highest number of articles in the field was published in 2017 (1,110; 11.65%). From 1994 to 2018, global publications in the field exhibited a strong trend for exponential growth (Figure 1c).

#### 3.1.3 Prediction of global publication trends

The logistic regression model was used to create the time curve of the number of publications from which future trends could be predicted (Figure 1d). This curve suggests that the field is currently in a phase of rapid growth in global publication outputs, but the rate of growth is expected to gradually decline within the next decade.

### 3.2 Quality of publications by country

#### 3.2.1 Total citation frequency

**Figure 2:**
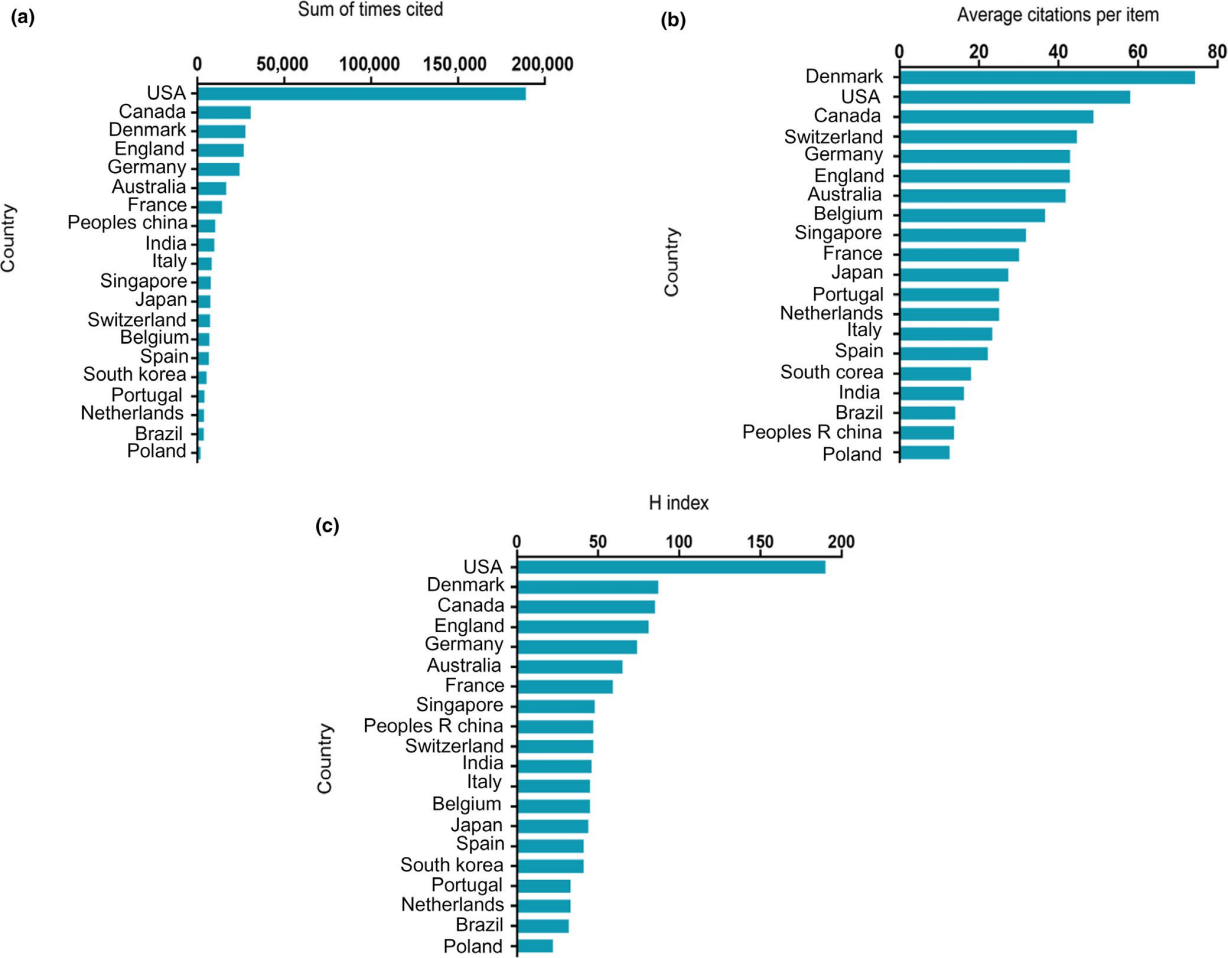
Citation frequency and H‐index of different countries publishing in *Pseudomonas aeruginosa* biofilm research. (a) Total citations of research articles from different countries. (b) Average citations per article from different countries. (c) H‐index of publications from different countries

Publications from the USA had by far the highest total number of citations (189,138), followed by Canada (30,633), Denmark (27,776), England (26,719), and Germany (24,281) (Figure 2a).

#### 3.2.2 Average citation frequency

Publications from Denmark had the highest average number of citations per publication (74.27), followed by the USA (58.00), Canada (48.78), Switzerland (44.61), and Germany (42.90) (Figure 2b).

#### 3.2.3 h‐index

Publications from the USA had the highest h‐index (190), followed by Denmark (87), Canada (85), England (81), and Germany (74) (Figure 2c).

### 3.3 Bibliographic coupling analysis

Bibliographic coupling measures links between publications that cite the same document. Two publications are “coupled” if they cite the same reference in their bibliographies, indicating the probability that these two publications share a common subject matter. The strength of a link between two publications indicates the number of cited references these two publications have in common. This can be scaled up to analyze the link strength of journals, institutions, and countries which have published papers within a particular subject matter, reflecting the degree of bibliographic coupling.

#### 3.3.1 Journals

**Figure 3:**
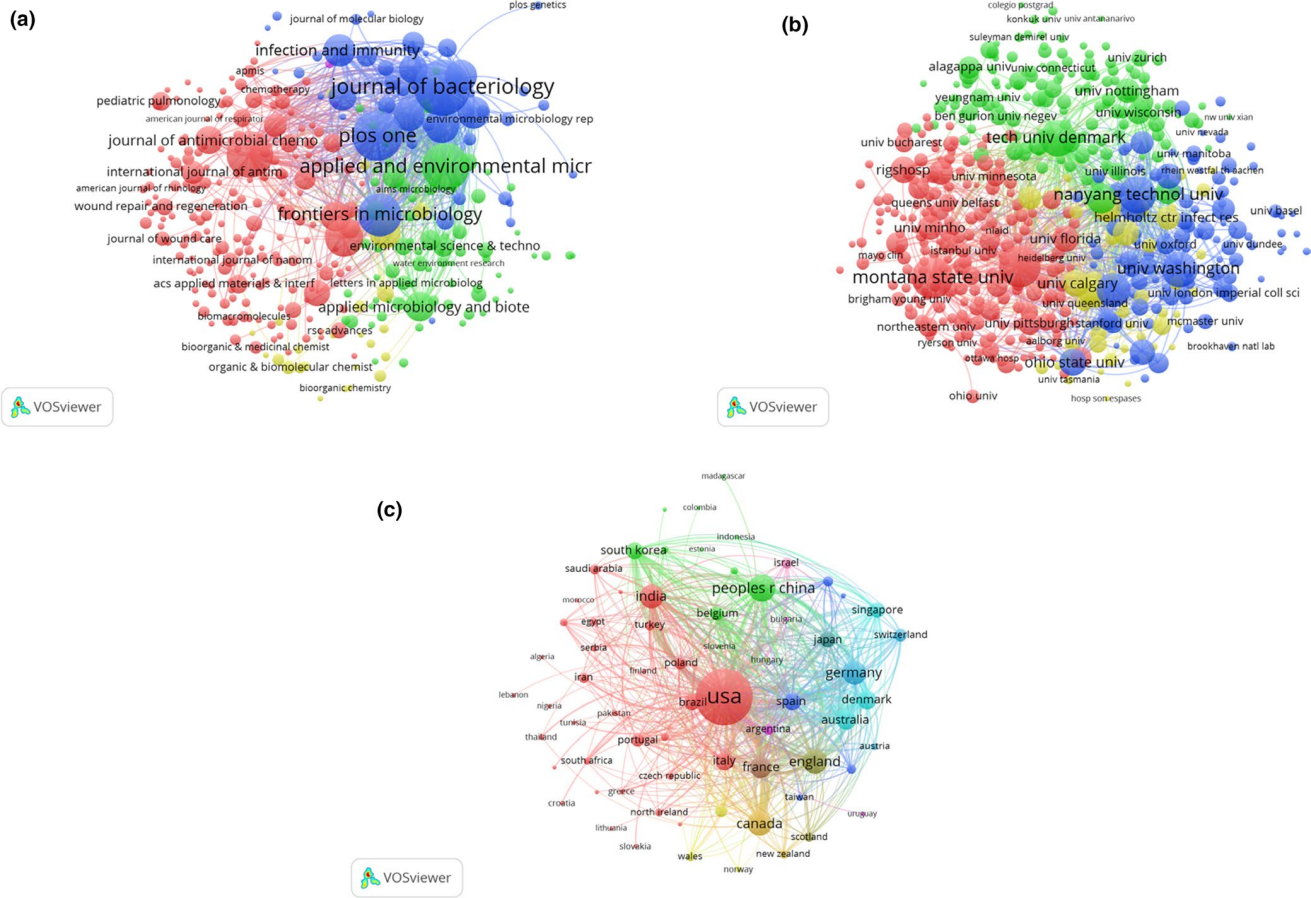
Bibliographic coupling analysis of global research on *Pseudomonas aeruginosa* biofilms. Mapping of (a) 332 identified journals, (b) 820 institutions, and (c) 68 countries in the research area. The line between two points in the map indicates that two journals/institutions/countries had established a similarity relationship. The thicker the line, the closer the link between the two entities

VOSviewer was used to analyze the journal names of all publications included in this study. Total link strength was shown for 332 identified journals (Figure 3a). The 5 journals with the highest total link strength were *Journal of Bacteriology* (2018 impact factor = 3.219; total link strength = 1,036,512 times), *PLoS ONE* (2.766; 571,946), *Applied and Environmental Microbiology* (3.633; 555,775), *Antimicrobial Agents and Chemotherapy* (4.256; 519,829), and *Frontiers in Microbiology* (4.019; 421,607).

#### 3.3.2 Institutions

VOSviewer was used to analyze the institutions of all publications included in this study. Total link strength was shown for 820 institutions, where each identified institution had a minimum of 5 publications in the field (Figure 3b). The 5 institutions with the highest total link strength were University of Copenhagen (total link strength = 1,362,392 times), Technical University of Denmark (1,109,331), Nanyang Technological University (905,333), Montana State University (882,748), and University of Washington (836,504).

#### 3.3.3 Countries

VOSviewer was used to analyze the countries of origin for all publications included in this study. Total link strength was shown for 68 countries, where each country had a minimum of 5 publications in the field (Figure 3c). The five countries with the highest total link strength were USA (total link strength = 7,981,965 times), Canada (1,991,097), Denmark (1,941,437), England (1,883,998), and Germany (1,864,856).

### 3.4 Coauthorship analysis

Coauthorship analysis measures publication links between researchers. The strength of a link between two researchers indicates the number of publications that they have coauthored. This can be used to analyze the link strength of individual authors or scaled up to reflect the coauthorship link strength of institutions and countries.

#### 3.4.1 Authors

**Figure 4:**
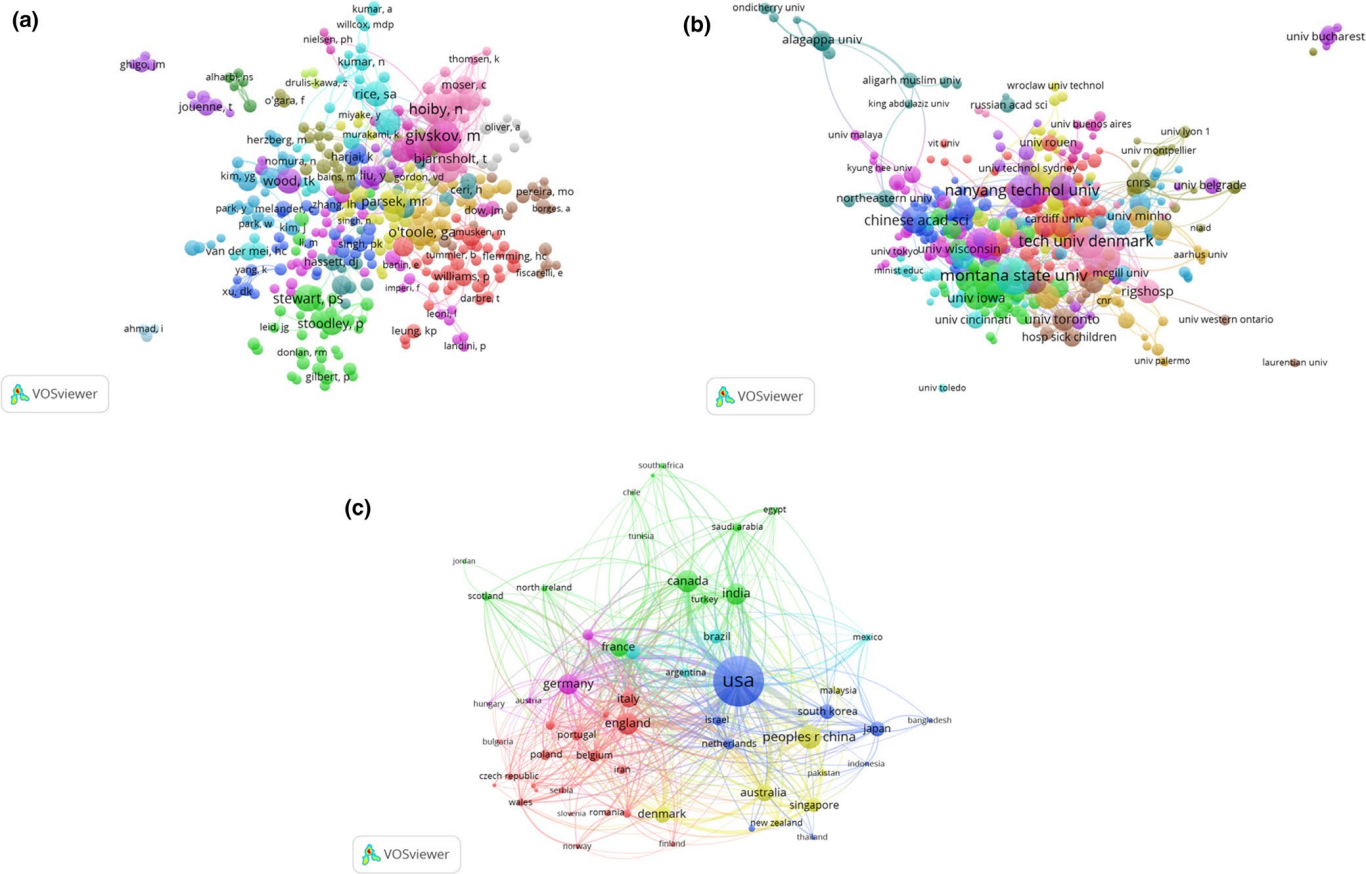
Coauthorship analysis of global research on *Pseudomonas aeruginosa* biofilms. Mapping of (a) 399 authors, (b) 960 institutions, and (c) 57 countries on the research area. The size of the points represents the coauthorship frequency. The line between two points indicates that two authors/institutions/countries had established collaboration. The thicker the line, the closer the collaboration between the two entities

VOSviewer was used to analyze a total of 399 authors with more than 10 publications in the field, identified from all of the publications included in this study (Figure 4a). The five authors with the highest total link strength were Givskov M (total link strength = 480 times), Høiby N (472), Bjarnsholt T (298), Jensen PO (289), and Tolker‐Nielsen T (244).

#### 3.4.2 Institutions

VOSviewer was used to analyze a total of 960 institutions with more than 10 publications in the field, identified from the publications included in this study (Figure 4b). The five institutions with the highest total link strength were University of Copenhagen (total link strength = 364 times), Nanyang Technological University (271), Technical University of Denmark (236), University of Washington (229), and Montana State University (169).

#### 3.4.3 Countries

VOSviewer was used to analyze a total of 57 countries with more than 10 publications in the field, identified from the publications included in this study (Figure 4c). The five countries with the highest total link strength were USA (total link strength = 1,247 times), England (530), Germany (401), Australia (354), and Canada (352).

### 3.5 Cocitation analysis

Cocitation analysis measures the link between two items that are both cited by the same document. Within a cocitation map of publications, the total link strength of a publication indicates the total number of cocitations the publication has with other publications included in the map. This can be scaled up to reflect the total cocitation link strength of authors and journals.

#### 3.5.1 Authors

**Figure 5:**
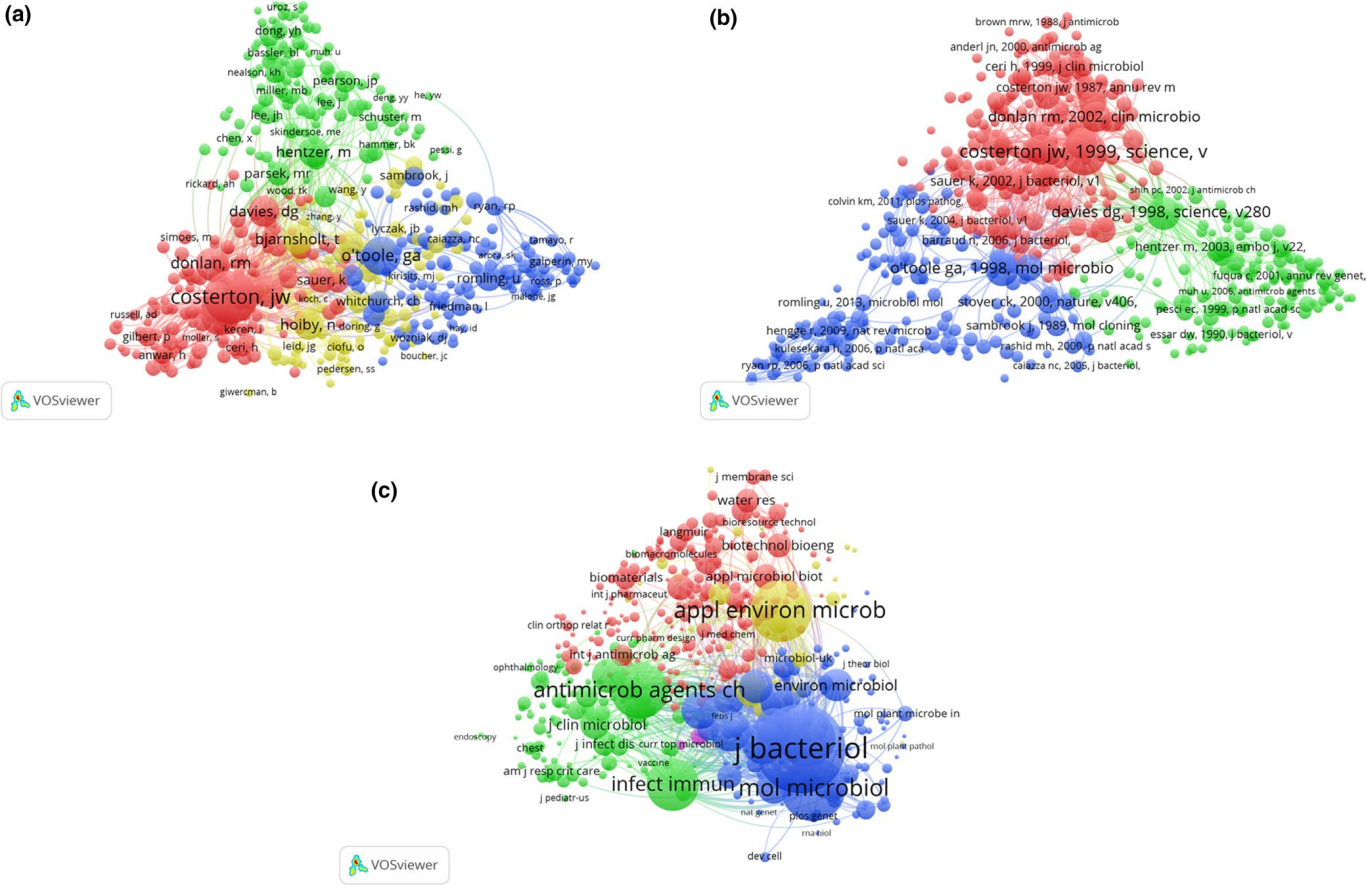
Cocitation analysis of global research on *Pseudomonas aeruginosa* biofilms. Points with the same color belong to the same research topic. (a) Mapping of cocited authors in the field. Points with different colors represent the 4,002 cited authors. (b) Mapping of cocited references in the field. Points with different colors represent the 3,103 cited references. (c) Mapping of cocited journals in the field. Points with different colors represent 1663 identified journals. The size of the points represents the citation frequency. A line between two points means that both were cited in one paper or journal. A shorter line indicates a closer link between two entities

VOSviewer was used to analyze a total of 4,002 authors that were cocited in more than 20 publications, identified from all of the publications included in this study (Figure 5a). The five authors with the highest total link strength were Costerton JW (total link strength = 77,314 times), Stewart PS (42,366), Hentzer M (40,329), O'Toole GA (39,113), and Davies DG (36,939).

#### References

3.5.2

VOSviewer was used to analyze a total of 3,103 references that were cocited in more than 20 publications, identified from all of the publications included in this study (Figure 5b). The five references with the highest total link strength were Costerton JW, 1996, *Science* (total link strength = 18,943 times); Davies DG, 1998, *Science* (15,070); O'Toole GA, 1998, *Mol Microbiol* (12,631); Costerton JW, 1995, *Annu Rev Microbiol* (9,985); and Sauer K, 2002, *J Bacteriol* (9,580).

#### 3.5.3 Journals

VOSviewer was used to analyze the journal names of the references for all publications included in this study, for which the journals needed to have more than 20 cocitations to be included in the analysis. The total cocitation link strength was shown for 1,663 identified journals (Figure 5c). The five journals with the highest total link strength were *Journal of Bacteriology* (total link strength = 2,385,391 times), *Molecular Microbiology* (1,366,686), *Applied and Environmental Microbiology* (1,199,218), *Proceedings of the National Academy of Sciences of the United States of America* (1,111,965), and *Antimicrobial Agents and Chemotherapy* (982,337).

### 3.6 Co‐occurrence analysis

**Figure 6:**
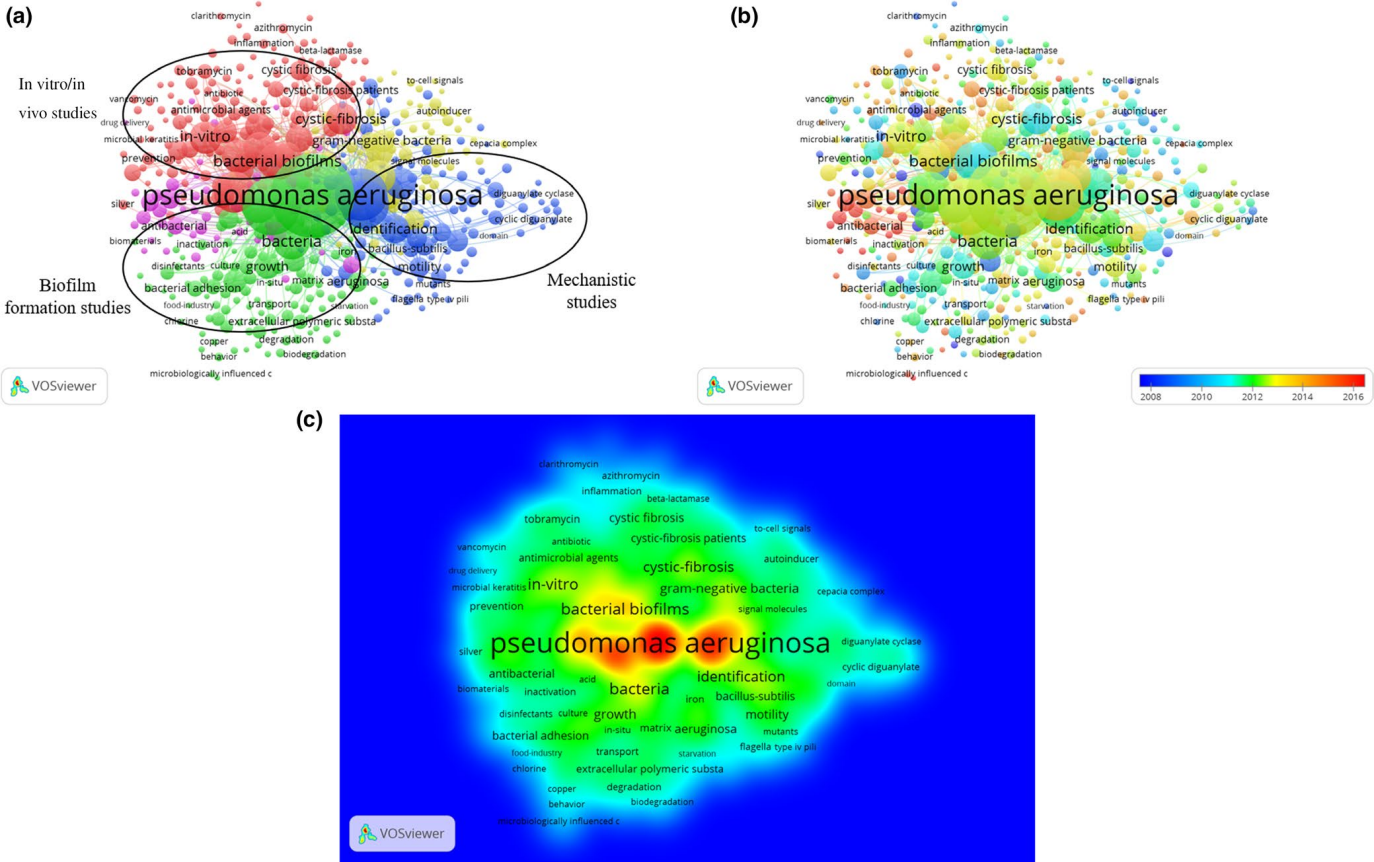
Co‐occurrence analysis of global research on *Pseudomonas aeruginosa* biofilms. (a) Mapping of keywords in the research area. The size of the points represents the frequency of appearance, and the keywords are divided into three clusters: in vitro/in vivo studies (red), biofilm formation studies (green), and mechanistic studies (blue). (b) Distribution of keywords according to the chronological order of appearance. Keywords in blue appeared earlier than those in yellow, and keywords in red appeared the latest. (c) Distribution of keywords according to the mean frequency of appearance. Keywords in red occurred with the highest frequency, followed by yellow, green, and cyan

Co‐occurrence analysis measures the co‐occurrence links between terms. The co‐occurrence link strength indicates the number of publications in which two terms occur together. Co‐occurrence analyses have been performed in scientific and other disciplines to monitor past developments and have proven useful in identifying future research directions and popular topics within a specific discipline. In this study, keywords were identified as words that have been used more than 5 times in titles and abstracts among all included publications, which were analyzed using VOSviewer. There were 2,825 keywords identified that were classified broadly into three clusters: “mechanistic studies,” “in vitro/in vivo studies,” and “biofilm formation studies” (Figure 6a). These clusters reflected the most prominent topics of research on *P. aeruginosa* biofilms to date.

The identified keywords were then defined by specific colors using VOSviewer based on the average time they appeared in all included publications (Figure 6b). Keywords that on average appeared during earlier time periods were denoted by colors toward the blue end of the spectrum, while keywords that on average appeared later were denoted by colors toward the red end. For the publications included in this study, most publications prior to 2014 appeared to focus on “mechanistic studies” and “in vitro/in vivo studies,” while “biofilm formation studies” appeared to be a topic attracting more attention in recent years and may become an important topic of focus in the near future. The same identified keywords were also mapped by frequency of appearance (Figure 6c).

## 4 DISCUSSION

### 4.1 Trends in the research field

In this study, we used a combination of bibliometric and visualized analyses to generate a representation of the current state of the field on *P. aeruginosa* biofilms research. We have analyzed the relative contributions of individual authors, journals, institutions, and countries to this well‐established but still rapidly progressing research field, as well as existing and evolving topic areas that are likely to attract continued research interest in the coming years. Since the field first picked up in 1994, there has been an exponential increase in publication output in the field leading up to 2018, with particularly dramatic increases seen over the past 3–4 years. This is due to the pronounced ability of *P. aeruginosa* to induce biofilm‐related disease that is causing significant and ongoing concern in the clinical setting, which has spurred increasing research effort in developing strategies that target their control and eradication (Qu et al., 2016). In our study, significant bibliographic coupling was found for publications arising from 820 institutions over 57 countries worldwide, among the almost ten thousand publications in the field that have appeared since 1994. These numbers reflect the strength of the field and the overwhelming knowledge that has been generated over the past 20 years. From the trends observed in this study, it is likely that *P. aeruginosa* biofilms will continue to be an epicenter of intense research interest in the future, possibly with more studies focusing on biofilm formation as indicated by our co‐occurrence analysis.

### 4.2 Quality of global publications by country, institution, and journal

#### 4.2.1 Countries

The USA is the world leader in the field of *P. aeruginosa* biofilm research, which had the highest total citation frequency and H‐index for its publications, as well as the top rank for bibliographic coupling and coauthorship analyses conducted by country. These trends suggest that publications from the USA collectively have the highest quality and academic impact in this field and also encompass the strongest collaborations worldwide. On the other hand, a number of European countries such as Denmark, England, Germany, and Switzerland have seen rapid growth in publication number, quality, and impact in this field over the past decade, as well as increasing collaboration with other countries.

The number of publications on *P. aeruginosa* biofilm research arising from China has increased rapidly over the past decade, as reflected by its total number of publications ranking second among countries worldwide. However, the total citation frequency, average citation frequency, and H‐index of these publications from China only ranked 8th, 19th, and 9th, respectively. This large discrepancy between the number of Chinese publications and their perceived quality and academic impact may be due to the inherent differences in the Chinese academic evaluation system compared with most Western countries, which focuses primarily on publication quantity rather than quality (Zhai, Wang, & Li, 2016). With increases in research funding in China over the recent years, the quality of publications from China should gradually improve, including in the field of *P. aeruginosa* biofilm research, and become more consistent with trends observed in global publications in the coming years.

#### 4.2.2 Institutions

The link strengths of institutions determined through bibliographic coupling and coauthorship analyses in our study reflected the relative contributions of specific institutions to the field of *P. aeruginosa* biofilm research. Unsurprisingly, the highest contributions to the field were from institutions based in the top contributing countries, particularly the USA and Denmark. Interestingly, within the top five ranks of bibliographic coupling and coauthorship analyses by the institution, the University of Copenhagen and Technical University of Denmark, which are both based in Denmark, were ranked higher than Montana State University and the University of Washington from the USA. Nanyang Technical University was the only institution whose home country (Singapore) was not ranked within the top 5 countries for total citation frequency, average citation frequency, and H‐index of publications in the field. These trends suggest that publication outputs from first‐class research institutes within the field of *P. aeruginosa* biofilm research can contribute significantly to the ranking of their respective home countries in the same field, but is not an absolute measure. The 5 top‐ranked institutions as indicated by bibliographic coupling and coauthorship analyses are likely to be the strongest in the field worldwide.

Coauthorship and cocitation analyses have also revealed Bjarnsholt T as the only author who was ranked within the top 5 for both of these analyses. Having generated publications from both the University of Copenhagen and Technical University of Denmark, this author has likely contributed to increasing the ranking of both institutions in the field of *P. aeruginosa* biofilm research.

#### 4.2.3 Journals

The link strengths of journals determined through bibliographic coupling and cocitation analyses in our study reflected the degree of relatedness of specific journals to the field of *P. aeruginosa* biofilm research and also gave an indirect indication of journal rankings in this field. In addition, cocitation analysis revealed that the landmark studies in *P. aeruginosa* biofilm research had the highest cocitation link strength, which also increased the field‐specific impact of the journals in which these studies were published. Combining the results of journal bibliographic and cocitation analyses, the *Journal of Bacteriology* ranks as the top journal that publishes the highest number of related articles in the field, followed by *Applied and Environmental Microbiology*, and *Antimicrobial Agents and Chemotherapy*.

### 4.3 Future outlook

Future directions in the field of *P. aeruginosa* biofilm research are indicated by co‐occurrence network maps clustered by topic area or publication date. Broad research directions in the field were identified as mechanistic studies, in vitro and in vivo studies, and biofilm formation studies, each of which encompassed a range of prominent subtopics (Figure 6a,c). Worth noting, however, are the more “remote” topic areas around the periphery of the visualization map, which do not yet belong within any of the large clusters and for which strong co‐occurrence links have not yet been established with other major topics in the field. These topic areas may include, for instance, biomaterials, fluid shear, and transcriptional activators. Although they do not represent mainstream research areas in the field, they do represent multidisciplinary approaches to a longstanding problem derived from other fields such as regenerative medicine, biomedical engineering, and molecular biology and warrant further investigations.

Information on topic areas in the field organized by publication date gives further interesting insights into future developments (Figure 6b). Several terms colored toward the red end of the spectrum, indicating more recent publication dates, belong under the “biofilm formation study” cluster, suggesting a shift in research direction to related topics in the coming years. More interestingly, some small areas of emerging research in the field also colored within the red spectrum due to their recent appearance in the literature, overlapped with the “remote” terms identified using the earlier co‐occurrence map grouped by topic area. These may represent rapidly evolving topics in the field of *P. aeruginosa* biofilm research where substantial research activity has already begun and will see significant developments in the near future, such as coatings, wounds, and multi‐drug resistance.

### 4.4 Strengths and limitations

We have conducted extensive bibliometric and visualized analyses to reflect the current state of *P. aeruginosa* biofilm research through analyses of citation frequency and quality, bibliographic coupling, coauthorship, cocitation, and co‐occurrence. This gives a large volume of information that reveals important trends through many different dimensions, which to the best of our knowledge is the first study of its kind in the field. Nevertheless, there were some limitations to the accuracy of our analyses. First, only English language studies from the WoS database were included in the analyses. The omission of non‐English language studies may have contributed to publication bias for language. Second, recently published studies may have had understated contributions to the different analyses due to their low citation frequency, despite some being published in high‐quality journals. The true impact of these recent publications and their influence on citation metrics of authors, journals, institutions, and countries in this field will only become more apparent with time. Third, country ranks reported in this study for the different types of analyses have not been adjusted for population, due to confounding factors such as the proportion of scientific researchers and research funding available in different countries. Due to these factors, adjusting the country ranking based on population may lead to additional bias. Nevertheless, the results of this study should be interpreted with a country's population in mind when comparing the contributions of countries with a large population such as the USA and China, to countries with a proportionally smaller population such as Denmark and Australia.

## 5 CONCLUSIONS

The present study shows the global trends in *P. aeruginosa* biofilm research based on bibliometric and visualized analyses of publication data. The USA and Denmark made the greatest contributions to this field, while the *Journal of Bacteriology* was the journal that produced the greatest number of relevant articles. The research effort in the field and publication outputs are likely to continue with an upward trend, particularly with heightened interest in recent years on expansion into multidisciplinary research areas. Continued research in the field of *P. aeruginosa* biofilms, particularly targeting multi‐drug‐resistant strains, will hopefully provide new solutions in the near future to address the longstanding clinical concerns they have caused.

## CONFLICT OF INTEREST

None declared.

## AUTHOR CONTRIBUTIONS

Yuanyuan Zhu: Conceptualization‐Equal, Data curation‐Equal, Formal analysis‐Equal, Writing‐original draft‐Supporting, Writing‐review & editing‐Supporting; Jiao Jiao Li: Conceptualization‐Equal, Formal analysis‐Equal, Investigation‐Equal, Writing‐original draft‐Lead, Writing‐review & editing‐Lead; Jian Ren: Data curation‐Equal, Investigation‐Equal, Methodology‐Equal, Writing‐review & editing‐Supporting; Siyang Wang: Data curation‐Equal, Investigation‐Equal, Methodology‐Equal, Writing‐review & editing‐Supporting; Ruiqin Zhang: Conceptualization‐Equal, Formal analysis‐Equal, Supervision‐Equal, Writing‐review & editing‐Equal; Bin Wang: Conceptualization‐Equal, Formal analysis‐Equal, Funding acquisition‐Lead, Supervision‐Equal, Writing‐original draft‐Supporting, Writing‐review & editing‐Equal.

## ETHICS STATEMENT

None required.

## Data Availability

The data analyzed during the current study are available in the Zenodo repository at https://doi.org/10.5281/zenodo.3662316.
